# Miller Fisher syndrome developing as a parainfectious manifestation of dengue fever: a case report and review of the literature

**DOI:** 10.1186/s13256-019-2066-z

**Published:** 2019-05-02

**Authors:** Nipun Lakshitha de Silva, Praveen Weeratunga, Thirugnanam Umapathi, Neelika Malavige, Thashi Chang

**Affiliations:** 1 Professorial Unit in Medicine, National Hospital of Sri Lanka, Colombo, Sri Lanka; 2 Department of Clinical Medicine, Faculty of Medicine, University of Colombo, Colombo, Sri Lanka; 3 Department of Neurology, National Neuroscience Institute, 11 Jln Tan Tock Seng, Singapore; 4 Centre for Dengue Research, University of Sri Jayewardenepura, Gangodawila, Sri Lanka

**Keywords:** Dengue, Guillain–Barré syndrome, Miller Fisher syndrome

## Abstract

**Background:**

Although dengue viral infections have emerged as one of the most important mosquito-borne diseases, neurological manifestations of dengue infections are uncommon. Guillain–Barré syndrome and Miller Fisher syndrome have been reported to occur as immune-mediated complications following dengue infection. We report the case of a patient who developed Miller Fisher syndrome during the acute phase of dengue fever suggesting that Miller Fisher syndrome may arise as a result of direct neurotropism of the dengue virus.

**Case presentation:**

A 70-year-old Sri Lankan man with well-controlled diabetes mellitus and hypertension presented with fever of 3 days’ duration, drooping of eyelids, dysarthria, and unsteady gait. He developed bilateral asymmetric partial ptosis, complete external ophthalmoplegia, bilateral palatal palsy, unilateral tongue weakness, ataxia, and areflexia from the second day of illness. He did not have limb weakness. He had evidence of acute dengue infection including progressive thrombocytopenia and leukopenia, positive dengue non-structural protein 1 antigen, dengue immunoglobulin M antibodies, and polymerase chain reaction detection of dengue virus genome in serum. Magnetic resonance imaging of his brain and cerebrospinal fluid analysis were normal. Polymerase chain reaction for dengue virus and immunoglobulin M antibodies in cerebrospinal fluid were negative. Nerve conduction studies showed axonal neuropathy. Antibodies (immunoglobulin G, immunoglobulin M, and immunoglobulin A) against GQ1b and GT1a were negative. He was treated with intravenously administered immunoglobulins and a recommended fluid regimen for dengue fever. He made a complete recovery from dengue fever in 7 days and Miller Fisher syndrome in 20 days.

**Conclusions:**

This case report highlights the rare occurrence of Miller Fisher syndrome during the acute phase of dengue fever. Neurological manifestations may occur as a consequence of direct neurotropism of dengue virus.

## Background

Dengue fever (DF) is the second commonest mosquito-borne infection after malaria [1]. It is estimated that approximately 390 million dengue infections occur annually worldwide out of which only approximately 90 million cases are clinically apparent [2]. Dengue viral infections have been endemic in Sri Lanka since the mid-1960s while epidemics of dengue hemorrhagic fever (DHF) have been recurring for almost three decades.

Dengue infection is often subclinical or present with a mild, undifferentiated, self-limiting acute febrile illness (DF). However, a proportion of patients with DF develop life-threatening disease characterized by plasma leakage and vascular shock (DHF).

Neurological manifestations in dengue infection have been recognized since the latter part of the twentieth century [3]. The pathophysiological basis of neurological manifestations in dengue infection remains not fully understood. Although encephalopathy and encephalitis are most frequently reported, the spectrum of neurological manifestations continues to expand from myositis, myelitis, cerebellitis, maculopathy, and other neuro-ophthalmic complications and mononeuropathies to Guillain–Barré syndrome (GBS) [4, 5]. GBS and its variants in dengue infection occur as immune-mediated complications 1 or more weeks after the acute infection [4, 6]. We report the case of a patient who developed Miller Fisher syndrome (MFS) during the acute phase of DF suggesting that the dengue virus may have a direct neurotropic effect.

## Case presentation

A 70-year-old Sri Lankan man with well-controlled diabetes mellitus and hypertension over 6 years developed acute onset, high-grade, intermittent fever associated with headache, arthralgia, myalgia, and nausea with no apparent focus of infection. On day 2 since onset of fever, he developed drooping of his eyelids and dysarthria. On day 3, he developed dysphagia and difficulty in walking because of unsteadiness. He did not experience any alteration of consciousness, seizures, sphincter dysfunction, limb weakness, or paresthesia. He was admitted to hospital on the third day of his illness. A timeline of the events starting from onset of fever is summarized in Table 1. There was no history of recent respiratory or gastrointestinal infection, or immunization. He had not had any neurological diseases in the past. His current medications included losartan for hypertension and metformin for diabetes mellitus.

**Table 1:** Timeline of events with diagnostic tests and interventions

Day of illness	Events	Diagnostic tests	Interventions
01	Onset of fever		
02	Right ptosis and dysarthria		
03	Bilateral asymmetric ptosis, complete ophthalmoplegia, mid-dilated pupils, swallowing difficulty due to palatal palsy, tongue deviation to right side, ataxia, and areflexia	Dengue NS-1 antigen positive Dengue RT-PCR positive	Admission to hospital, monitoring and fluid management for dengue fever
04		Non-contrast CT (brain) – normal	Intravenously administered polyclonal immunoglobulin started, supportive therapy
06	Improvement of ophthalmoplegia and ptosis, resolution of fever	MRI (brain) – normal Nerve conduction studies – evidence of mild axonal polyneuropathy	
07		Dengue immunoglobulin M (serum) – positive	
08	Improvement of ataxia, ability to walk without support		Intravenously administered polyclonal immunoglobulin (5 days) completed
12		CSF studies – pus cells – nil, lymphocytes 2/μl, protein 20 mg/dl, sugar 80 mg/dl (random blood sugar 131 mg/dl)	
13			Discharged from ward
20	Clinic review – complete neurological resolution		Discharged from clinic

*CSF* cerebrospinal fluid, *CT* computed tomography, *MRI* magnetic resonance imaging, *NS-1* non-structural protein 1, *RT-PCR* reverse transcriptase-polymerase chain reaction

On examination, his body temperature was 38.5 °C while general examination and respiratory, cardiovascular, and abdominal examinations were normal. His heart rate was 76 beats per minute and his blood pressure was 140/90 mmHg. On neurological examination, he was noted to be conscious, alert, and oriented. He had bilateral asymmetric ptosis more on right side, mid-dilated pupils with sluggish reaction to light, and complete bilateral external ophthalmoplegia but without diplopia; optic fundi, visual fields, and acuity were normal. He had bilateral palatal weakness and tongue deviation to right side; the rest of his cranial nerves were normal. He had a broad-based ataxic gait, dysdiadochokinesia, and dysmetria; all tendon reflexes were absent; the rest of the neurological examination of limbs, including sensation, was normal.

Investigations revealed thrombocytopenia with a platelet count of 106 × 10^9^/l on day 3, which dropped further to 17 × 10^9^/l on day 6. His platelet count then gradually increased to 164 × 10^9^/l by day 13. His white cell count reduced to 4200 × 10^9^/l on day 5 and then gradually increased to 7100 × 10^9^/l on day 13. Hematocrit was 40% and stable throughout the course of the illness. His creatinine was 99 μmol/l; serum sodium 132 mmol/l; and potassium 3.6 mmol/l. Serum aspartate aminotransferase (AST) showed a rise from 115 U/l on day 3 to 243 U/l on day 5 and normalized to 43 U/l by day 10. Alanine aminotransferase (ALT) was 55 U/l on day 3, increased to 127 U/l on day 5, and normalized to 37 U/l by day 10. Other liver functions were normal. His erythrocyte sedimentation rate was 18 in the first hour and C-reactive protein was 32 mg/l. Urine analysis and an ultrasound scan of his abdomen were normal.

A dengue non-structural protein 1 (NS-1) antigen test by rapid diagnostic test and real-time reverse transcriptase-polymerase chain reaction (RT-PCR) done on the third day of illness and dengue IgM antibodies by enzyme-linked immunosorbent assay (ELISA) tested on the seventh day of illness were positive. Serum IgM antibodies to West Nile virus and Japanese encephalitis virus by ELISA were negative on day 7. Nerve conduction studies showed evidence of mild axonal polyneuropathy. Repetitive nerve stimulation did not show decrement. Computed tomography (CT) and magnetic resonance imaging (MRI) scans of his brain were normal. Cerebrospinal fluid (CSF) analysis performed on the 12th day of illness after recovery of thrombocytopenia was normal with no albuminocytologic dissociation. PCR for dengue virus and dengue IgM antibodies in CSF were negative. Antibodies (IgG, IgM, and IgA) against a panel of gangliosides including GQ1b and GT1a were negative.

DF was treated with fluid replacement at 100 ml/hour while monitoring for plasma leakage clinically and ultrasonically. His fever subsided after 5 days from onset and all hematological parameters returned to normal subsequently. He was treated with intravenously administered immunoglobulin 0.4 g/kg for 5 days starting from the fourth day of his illness. He required nasogastric feeding because of dysphagia. He was treated with swallowing and speech therapy, and gait and balance training.

From around the sixth day of illness, his ptosis and ophthalmoplegia began to improve gradually. His ataxia improved enabling him to walk without support from the eighth day onward. He was discharged from hospital on the 13th day of illness and continued nasogastric feeding, physiotherapy, and speech therapy at home. At review 1 week later, he had made a complete neurological recovery with normal swallowing, complete eye movements, normal gait, and re-emerged deep tendon reflexes.

## Discussion and conclusions

Acute onset of the triad of ophthalmoplegia, areflexia, and ataxia defines the syndrome of MFS. Although not typical, bulbar paralysis has been reported in up to 60% of patients with MFS [7–9]. The constellation of symptoms and signs in our patient was consistent with MFS. Among the differential diagnoses considered, myasthenia gravis was ruled out on the basis of pupillary involvement, areflexia, and lack of fatigability while Bickerstaff brainstem encephalitis was ruled out based on the lack of upper motor neuron signs, intact sensorium, and normal brain images on MRI. Furthermore, an essentially normal nerve conduction study, apart from mild axonal polyneuropathy related to longstanding diabetes, is consistent with MFS [10]. Although the typical CSF finding in GBS describes albuminocytologic dissociation, normal protein levels have been reported in MFS [11]. Serum anti-GQ1b antibodies are highly sensitive and confirmatory of a clinical diagnosis of MFS [10], but were negative in our patient supporting the hypothesis of direct viral neurotropism rather than immune-mediated injury.

Although rare, GBS has been reported as a neurological manifestation of dengue infection [4, 6]. It usually occurs after the acute phase of the dengue infection suggesting an immunological basis for its manifestation. Reports of concomitant onset of both dengue and GBS are scarce. In a case series from Rio de Janeiro, out of seven patients with GBS who were positive for dengue IgM, one patient had concomitant dengue infection and GBS. However, available clinical information does not provide convincing evidence for the diagnosis of DF while the authors claimed that they were unable to exclude other infections [12]. There are a few other case reports of GBS occurring during DF [13, 14]. In contrast, there is only one case report of probable MFS associated with DF, but this could not be critically appraised due to the unavailability of English text of the article [15]. Our patient did not have any preceding infection or vaccination to suggest the usual immune-mediated basis for MFS. Negative PCR assays and IgM antibodies in CSF does not exclude acute CNS infection by dengue virus since these tests can be negative in many owing to low viral loads and low sensitivity of IgM in CSF [6]. A lumbar puncture had to be delayed until recovery of thrombocytopenia. Zika virus has been reported to cause GBS as a parainfectious manifestation more than as a postinfectious consequence [16]. The mechanisms postulated in the development of GBS in Zika virus infection include immune-mediated molecular mimicry during the incubation period, direct viral neuropathogenic effects, a hyperacute immune response, or immune mechanisms other than molecular mimicry [17]. It is plausible that similar mechanisms occur when GBS or MFS occur as a parainfectious manifestation of dengue infection. There is evidence that dengue virus can actively penetrate the blood–brain barrier to enter the central nervous system [16]. Neurotropism of dengue virus is well recognized [4] in causing dengue encephalitis, but there have not been studies so far to demonstrate dengue virus in the peripheral nervous system.

This case report highlights the potential for neurotropism of dengue virus in the peripheral nervous system to cause MFS as a parainfectious rather than a postinfectious manifestation of flaviviral infection.

## Abbreviations

ALT: Alanine aminotransferase
AST: Aspartate aminotransferase
CSF: Cerebrospinal fluid
CT: Computed tomography
DF: Dengue fever
DHF: Dengue hemorrhagic fever
ELISA: Enzyme-linked immunosorbent assay
GBS: Guillain–Barré syndrome
MFS: Miller Fisher syndrome
MRI: Magnetic resonance imaging
PCR: Polymerase chain reaction
RT: Reverse transcriptase

## Authors’ information

Dr Nipun Lakshitha de Silva

Postgraduate trainee in clinical medicine at the Professorial Unit in Medicine, National Hospital of Sri Lanka and lecturer (probationary) in Clinical Medicine, Faculty of Medicine, General Sir John Kotelawala Defence University.

Dr Praveen Weeratunga

Lecturer in Medicine, Faculty of Medicine, University of Colombo

Senior Registrar in Medicine, Professorial Medical Unit, National Hospital of Sri Lanka

Thirugnanam Umapathi

Senior Consultant Neurologist, National Neuroscience Institute, Singapore

Neelika Malavige

Director, Centre for Dengue Research, University of Sri Jayewardenepura

Prof. Thashi Chang

Professor in Neurology in the Department of Clinical Medicine at the University of Colombo, Sri Lanka and honorary Consultant Neurologist at the Professorial Unit in Medicine, National Hospital of Sri Lanka
